# Combined Effects of Curcumin and Carboplatin on Mitochondrial Depolarization and Caspase Activation in Human Lung Adenocarcinoma Cells

**DOI:** 10.2174/0118715206360549241209111501

**Published:** 2025-05-15

**Authors:** Yüksel Öğünç Keçeci, Mine Ezer Oktay, Zerrin Incesu, Filiz Özdemir

**Affiliations:** 1 Department of Biochemistry, Faculty of Pharmacy, Anadolu University, Eskişehir, Turkey;; 2 Tekgen Healthcare Services CanCell Precision Oncology Solution Center, İstanbul, Turkey

**Keywords:** Curcumin, carboplatin, mitochondrial apoptosis, caspase activation, annexin V-FITC labeling method, caspase 9 activity

## Abstract

**Background:**

In various kinds of cancer, including Non-Small Cell Lung Cancer (NSCLC), treatment resistance diminishes the effectiveness of current therapeutic approaches and underscores the need for new treatment strategies.

**Aims:**

This study aimed to investigate the combined and individual effects of the anticancer drug carboplatin and the natural antioxidant curcumin, as well as the apoptotic effects of these drugs on the A549 cancer cells.

**Objectives:**

The synergistic effect of the combined treatment with curcumin and carboplatin on lung cancer cells was evaluated, focusing on early apoptosis, caspase-3/9 activity, and mitochondrial membrane potential.

**Methods:**

The cytotoxic effects were determined using the MTT method. Apoptotic changes were examined using the Annexin V-FITC labeling method. Activation of caspases-9 and -3 and mitochondrial membrane potential were measured using flow cytometry.

**Results:**

The IC_50_ values of curcumin and carboplatin against A549 cells were determined to be 60±8 μM and 100±9 μM, respectively. The combination of curcumin and carboplatin showed a synergistic effect. After treating A549 cells with carboplatin, curcumin, or the combined use of curcumin+carboplatin for 12 hours, the rates of early apoptotic cells were determined to be 9.5±1.3%, 8.1±0.3%, and 22.2±2.9%, respectively. The rate of early apoptosis in combined use was significantly higher compared to individual use. Similarly, when the combined treatment of curcumin and carboplatin was compared to the administration of carboplatin alone, a higher level of mitochondrial membrane depolarization was observed. There was a slight increase in caspase 9 activity in the combined treatment group compared to the individual treatments. Furthermore, after treating A549 cells with the specified doses, the caspase 3 activity was determined for carboplatin (0.5±0.1%), curcumin (1.9±0.0%), and the combination of both (7.3±0.8%).

**Conclusion:**

These results indicated that the combined use of curcumin and carboplatin enhanced apoptosis and mitochondrial depolarization, demonstrating that the combined treatment of drugs reduced the toxic dose of carboplatin. However, further research is needed to comprehensively understand the potential of this effect in *in vivo* studies.

## 1 INTRODUCTION

The second most common cause of mortality worldwide is cancer [1, 2]. Lung carcinoma, especially Non-Small Cell Lung Cancer (NSCLC), is a very dangerous and prevalent disease [3], constituting 80-85% of lung cancers [4]. A total of 18.0% of cancer-related deaths and 11.4% of cancer diagnoses are caused by lung cancer [5]. Every year, nearly 1.8 million individuals lose their lives to lung cancer [6].

Chemotherapy is frequently used to treat cancer. However, it has been reported to potentially cause drug resistance due to the high heterogeneity of cancer cells [7, 8]. To solve this problem, various chemotherapeutics can be used to prevent tumor metastasis, cell proliferation, and invasion [9, 10]. Combination or multicomponent therapies using different chemotherapy drugs are employed not only to enhance the effects of drugs but also to reduce the toxicity induced by these drugs [11]. Platinum drugs are used alone or in combination with additional drugs to treat a variety of human cancer types [12-16]. However, the development of chemotherapy resistance to platinum drugs in almost all NSCLC patients underscores the need to find new single or combination therapies [17-19].

Research on platinum drugs has spanned approximately forty years; however, the forms of circulating agents, mechanisms of cell entry, and primary events leading to drug-induced cell death are not fully understood [20, 21]. Carboplatin is a second-generation Pt^2+^ anticancer drug widely utilized in clinical settings for managing lung, ovarian, and various cancer types. Although carboplatin shares mechanisms of action similar to cisplatin, it exhibits considerably lower toxicity than cisplatin [12]. Curcumin, a polyphenol present in turmeric, is also known as diferuloylmethane [22]. It has been reported to have antioxidant, anti-inflammatory [23], antimutagenic, antimicrobial [24, 25], and anticancer properties [26, 27]. Curcumin can suppress the proliferation of A549 cells and trigger apoptosis [28, 29]. It is well known that curcumin shields healthy organs from the harmful effects of chemotherapy while also sensitizing cancer cells to a variety of chemotherapeutic drugs [30]. Studies have shown that curcumin can increase the sensitivity of lung cancer cells to various chemotherapeutic drugs, such as vinorelbine, cisplatin, docetaxel, and gemcitabine [31-34].

In this study, the synergistic effect of curcumin and carboplatin combination on apoptotic-related proteins in lung cancer cells was investigated, and the apoptotic signaling pathway was elucidated.

## 2 MATERIALS AND METHODS

### 2.1 Cell Culture and Reagents

The A549 cell line used in this study was obtained from the American Type Culture Collection (ATCC^®^, catalog number CCL-185).

A549 cancer cells were grown in a medium containing 10% fetal bovine serum, 1% penicillin/streptomycin, and RPMI 1640 (with L-glutamine, Sigma Aldrich-UK) at 37°C in an incubator with an internal atmosphere of 5% CO_2_. Every two or three days, the cells were divided into subcultures using a 1:3 ratio after being treated with trypsin/EDTA solution. We purchased carboplatin and curcumin from Sigma Aldrich, UK.

### 2.2 Cytotoxicity Assay

First, different concentrations of carboplatin (2.5, 5, 10, 25, 50, 75, 100, and 200 µM) and curcumin (2.5, 5, 10, 20, 40, 60, 80, 100, and 150 µM) doses were prepared, and the IC_50_ value was determined using the MTT [3-[4,5-dimethythiazol-2-yl]-2,5–diphenyltetrazolium bromide] method [35]. Later, combinations of carboplatin and curcumin were created using different percentages of IC_50_ doses (100%, 75%, 50%, 40%, 30%, 20%, and 10%). The IC_50_ value of these carboplatin-curcumin combinations on A549 cells was determined using the MTT method. Briefly, 0.5 mg/ml of MTT solution was added to each well, and the mixture was then incubated for 3 hours at 37°C. Using a microplate reader, the absorbance was measured at 540 nm after the insoluble purple formazan product was dissolved in DMSO.

### 2.3 Flow Cytometric Analysis of Cellular Apoptosis

The detection of apoptotic cells was carried out using the FITC Annexin V Apoptosis Detection Kit II (BD Pharmingen) in accordance with the manufacturer's instructions. A549 cells were treated with curcumin (60 μM), carboplatin (100 μM), and a combination of curcumin+carboplatin (24 μM +40 μM and 18 μM +30 μM) for 6 and 12 hours. Following the transfer of the cells into tubes with 1 ml of media, the centrifuge was run for 5 minutes at 1400 rpm. Following centrifugation, 100 μl of binding buffer was pipetted into each tube after the supernatant in the tubes was decanted. Each tube was filled with Annexin V-FITC and propidium iodide (PI) dye, except for the negative control group, which did not receive any dye. The cells were then left at room temperature in the dark. After 15 minutes, binding buffer was added to the tubes, and they were analyzed with a flow cytometer (Becton-Dickinson FACS).

### 2.4 Mitochondrial Membrane Potential Assessment

The flow cytometry mitochondrial potential detection kit (BD) was used to detect mitochondrial membrane potential in accordance with the manufacturer's instructions. The cells were centrifuged, and the supernatant was discarded. After adding 0.5 ml of JC-1 working solution, they were incubated for 15 minutes in a CO_2_ incubator. Following incubation, the cells were washed with assay buffer. The cells underwent a 5-minute centrifugation at 400×g. Every cell pellet was centrifuged and then carefully resuspended in assay buffer before being examined using a flow cytometer (Becton-Dickinson FACS Aria, USA).

### 2.5 Caspase-9 Activity Assay

The detection of caspase-9 activity was carried out using the CaspGLOW Fluorescein Active Caspase 9 Staining Kit (Thermo). The cells were centrifuged, and then 300 μl of cell media was added to each sample. The culture tubes were filled with 1 μl of FITC-LEHD-FMK and incubated for 1 hour at 37°C in an incubator with 5% CO_2_. They were centrifuged for three minutes at 3000 rpm at the conclusion of the incubation period. Each sample was mixed with a washing solution and centrifuged after discarding the supernatant portion. Following the disposal of the supernatant, the samples were suspended in a washing solution and then examined using flow cytometry.

### 2.6 Caspase-3 Activity Assay

The detection of caspase-3 activity was carried out using the FITC Active Caspase-3 apoptosis kit (BD). After centrifuging the cell suspensions, cold PBS was used to wash the pellets. Then, the cells were treated for 20 minutes on ice in the dark with 0.5 ml of Cytofix/Cytoperm solution. A washing solution was used to wash the pellets. The cells were suspended in 100 µl of washing solution and 20 µl of caspase-3 antibody, followed by a 30-minute incubation period in the dark at room temperature. The cell pellets were then washed and after the disposal of the supernatant, the samples were suspended in a washing solution and then examined using flow cytometry.

### 2.7 Statistical Analysis

The experiments were repeated three times. The results were calculated assuming the viability values of the control groups to be 100% in the MTT assay, and statistical analysis was performed using the SPSS Statistics 17 program.

## 3 RESULTS

### 3.1 Cell Viability Assay and Combination Index (CI)

**Fig. (1):**
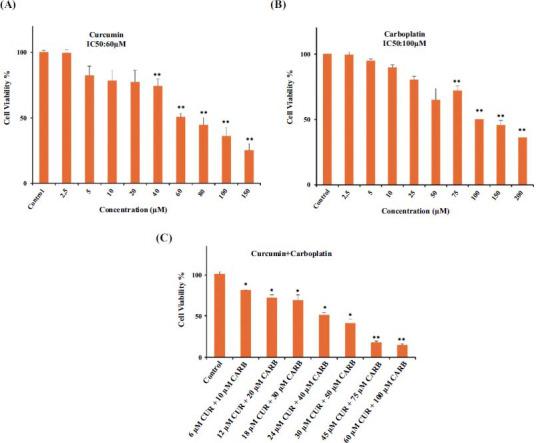
The cell viability of A549 cells treated with curcumin (**A**), carboplatin (**B**), and a combination of curcumin+carboplatin (CUR+CARB) (**C**). *p* < 0.05 was taken as criteria to denote statistical significance in all tests and statistically significant differences were shown by (*: *p* < 0.05; **: *p* < 0.01). Each experiment was repeated 3 times independently.

The IC_50_ values of curcumin and carboplatin against A549 cells were determined as 60±8 μM and 100±9 μM, respectively, for 24 hours (Figs. **1A** and **B**). Combination doses were prepared according to IC_50_ doses of curcumin and carboplatin. The combination of 24 μM curcumin + 40 µM carboplatin and 18 μM curcumin + 30 μM carboplatin, which were 40% and 30% of the IC_50_ values, inhibited cell proliferation by 48.9% and 30.9%, respectively (Fig. **1C**, Table **1**). These two combinations were selected to continue the experiment [36].

### 3.2 Determination of Early and Late Apoptotic Cells

**Fig. (2):**
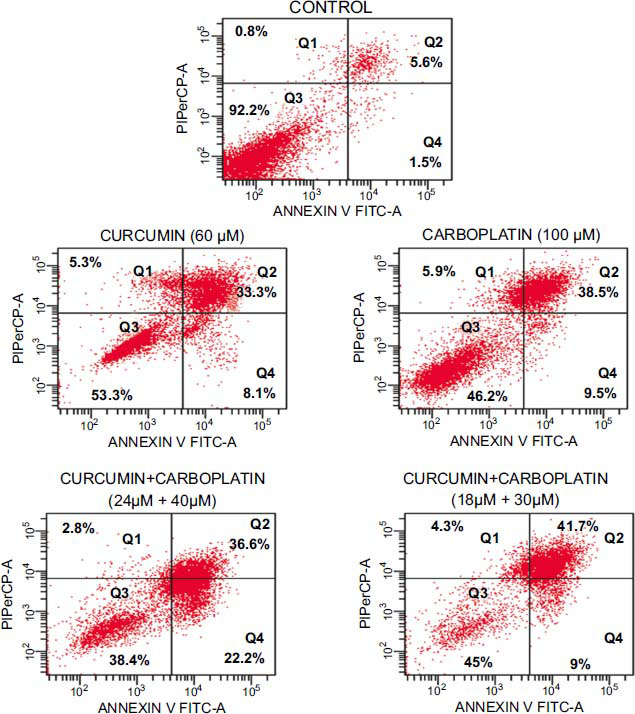
The early and late apoptosis analysis results of A549 cells incubated for 12 hours with control, curcumin, carboplatin, and curcumin+carboplatin combination groups. Necrotic cells (Annexin-V͞ /PI+ Q1), late apoptotic (Annexin-V+ /PI+, Q2), viable (Annexin-^V͞^ /P^I͞^, Q3), and early apoptotic (Annexin-V^+^ /P^I͞^, Q4).

After treating A549 cells with an IC_50_ dose of carboplatin (100 µM), an IC_50_ dose of curcumin (60 µM), and combined use of curcumin+carboplatin (24 µM+40 µM) for 12 hours, the percentages of early apoptotic cells were determined as 9.5±1.3%, 8.1±0.3%, and 22.2±2.9%, respectively. The rate of early apoptosis in combined use was found to be higher when compared to individual use (Fig. **2**, Tables **2** and **3**).

### 3.3 Determination of Mitochondrial Membrane Potential

**Fig. (3):**
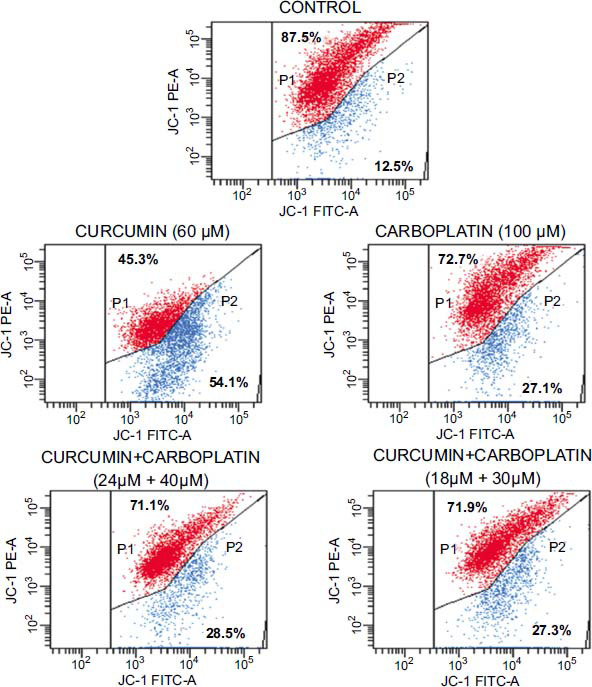
The mitochondrial membrane potential analysis results of A549 cells incubated for 12 hours with control, curcumin, carboplatin, and curcumin+carboplatin combination groups.

After treating A549 cells with an IC_50_ dose of carboplatin (100 µM), an IC_50_ dose of curcumin (60 µM), and combined use of curcumin+carboplatin (24 µM+40 µM) for 12 hours, the percentages of mitochondrial membrane depolarization were determined as 27.1±3.2%, 54.1±6.5%, and 28.5±4.7%, respectively (Fig. **3** and Table **4**). After treating A549 cells with an IC_50_ dose of carboplatin (100 µM), an IC_50_ dose of curcumin (60 µM), and combined use of curcumin+carboplatin (24 µM+40 µM) for 24 hours, the percentages of mitochondrial membrane depolarization were determined as 16.8±0.0%, 54.6±18.9%, and 29.5±1.5%, respectively (Table **4**). The results obtained showed that curcumin was highly effective on mitochondrial membrane potential, and when combined doses of curcumin and carboplatin were used, the apoptotic effect was found at the lowest dose of carboplatin compared to IC_50_ doses of carboplatin.

### 3.4 Determination of Caspase 9 Activity by Flow Cytometry

**Fig. (4):**
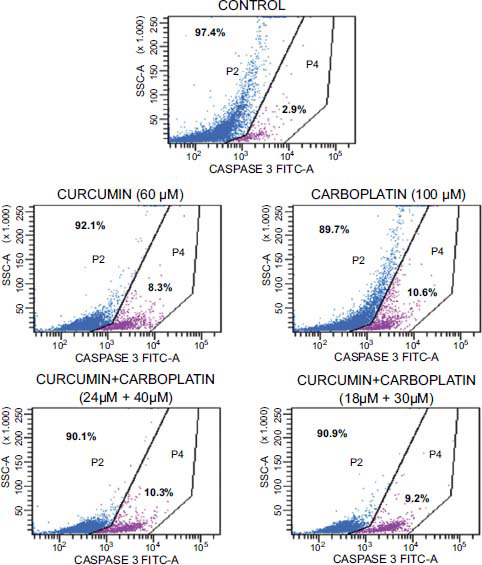
The caspase 9 activity analysis results of A549 cells incubated for 12 hours with control, curcumin, carboplatin, and curcumin+carboplatin combination groups.

After treating A549 cells with an IC_50_ dose of carboplatin (100 µM), an IC_50_ dose of curcumin (60 µM), and combined use of curcumin+carboplatin (24µM+40 µM) for 12 hours, the caspase 9 activity was determined as 10.6±2.0%, 8.31±0.8%, and 10.3±1.4%, respectively (Table **5** and Fig. **4**). The combined treatment group exhibited a slight increase in caspase 9 activity compared to the individual treatments.

### 3.5 Determination of Caspase 3 Activity by Flow Cytometry

After treating A549 cells with an IC_50_ dose of carboplatin (100 µM), an IC_50_ dose of curcumin (60 µM), and combined use of curcumin+carboplatin (24 µM+40 µM) for 12 hours, the caspase 3 activity was determined as 0.7±0.7%, 1.6±0.4%, and 1.6±0.1%, respectively. After treating A549 cells with an IC_50_ dose of carboplatin (100 µM), an IC_50_ dose of curcumin (60 µM), and combined use of curcumin+carboplatin (24 µM+40 µM) for 24 hours, the caspase 3 activity was determined as 0.5±0.1%, 1.9±0.0%, and 7.3±0.8%, respectively. Caspase 3 activity was found to be higher in the combined treatment group compared to the individual treatments at 24 hours (Table **6**).

## 4 DISCUSSION

Chemotherapy is generally considered the most appropriate treatment approach for Non-Small Cell Lung Cancer (NSCLC) [37]. It involves the administration of chemical agents aimed at inhibiting the growth of cancer cells and inducing their death through cytotoxic effects [38].

Carboplatin and cisplatin are two of the most commonly used platinum-based chemotherapeutics for lung cancer. Carboplatin exerts an antineoplastic effect by inhibiting DNA and RNA polymerase without repairing nucleotides. Studies have demonstrated that carboplatin disrupts cell division, growth, and proliferation by altering the structure of DNA nucleosomes [39]. It has been revealed in some studies that carboplatin causes DNA damage in cells due to its platinum content and thus inhibits the development and spread of tumor cells in the body [40]. The effects of chemotherapy also cause serious damage to normal cells. DNA damage comes first among these damages. New methods are being developed to minimize this damage. Recent studies have focused on phytochemicals as anticancer agents. Dietary antioxidants are recommended as therapeutic agents to prevent cellular damage. It is known that dietary antioxidants shrink tumor cells, repair cellular damage, and eliminate the harmful effects of reactive oxygen species. It has also been stated in many studies that normal cells maintain integrity [41-43]. Curcumin exhibits therapeutic potential against various cancer types, including skin, breast, lung, liver, and colon cancers [44, 45].

A549 cells were treated with different dosages of curcumin and carboplatin alone and in combination at various ratios for 24 hours in order to examine the synergy between these two drugs (Fig. **1**). The MTT assay demonstrated a dose-dependent reduction in cell viability caused by both curcumin and carboplatin alone and in combination. The IC_50_ dose of carboplatin was determined as 100 µM, and the IC_50_ dose of curcumin was determined as 60 µM. Cell proliferation was suppressed by 48.9% and 30.9%, respectively, by the combination of 24 μM curcumin + 40 µM carboplatin and 18 μM curcumin + 30 μM carboplatin, which were 40% and 30% of IC_50_ values. The combination index was calculated using the CompuSyn program. This calculation can be used to determine whether the drugs have a synergistic (CI<1), additive (CI=1), or antagonistic (CI>1) effect [46, 47]. According to these results, the combination of these two agents (24 μM curcumin + 40 µM carboplatin) and (18 µM curcumin + 30 µM carboplatin) demonstrated CI <1, and they were thus determined to have a synergistic effect. Due to these results, subsequent experiments were conducted using these concentrations. Similar to our study, Kang *et al*. (2015) stated that the combination of curcumin and carboplatin had a synergistic effect on A549 cells [48].

Apoptosis, a tightly controlled cell death mediated by many genetic and pharmacological processes, is essential for appropriate tissue growth and homeostasis. Defects in the mechanism of apoptosis are crucial to the development of tumors because they prevent external survival factors, prolong the life of neoplastic cells, and shield them from hypoxia and oxidative stress as the tumor mass grows [49].

After treating A549 cells with carboplatin (100 µM), curcumin (60 µM), and combined use of curcumin+carboplatin (24 µM+40 µM), the percentages of early apoptotic cells were determined to be 9.5±1.3%, 8.1±0.3%, and 22.2±2.9%, respectively. According to this result, co-treatment with carboplatin used as an anti-neoplastic drug and curcumin, which has a known antioxidant effect, on the A549 cancer cell line increased the rate of early apoptosis and reduced the toxic doses of carboplatin.

In one study, it was reported that treatment using curcumin with cisplatin, which is a platinum-group drug, increased the rate of apoptosis of bladder cancer cells by triggering ROS production and upregulating the p-ERK1/2 and caspase-3 activation [50]. Consistent with our study, Ma *et al*. reported that H838 and H520 cell lines were treated with cisplatin and resveratrol, a natural polyphenol compound similar to curcumin, individually and in combination. It was reported that compared to the control group, there was a considerable increase in early apoptotic cells in both cancer cell lines, and the combination treatment led to more apoptosis compared to a single treatment [51].

Mitochondrial membrane depolarization (ΔΨm) is a key indicator of the mitochondrial pathway of apoptosis. When ΔΨm is lost, proapoptotic factors are released into the cytoplasm, which is followed by the apoptotic process based on caspase activation [52]. Many studies have assessed mitochondrial membrane potential utilizing flow cytometry. In the present study, JC-1 dye was used to examine the integrity of the mitochondrial membrane. When ΔΨm in apoptotic cells was disrupted, the JC-1 dye turned green. Alterations in mitochondrial membrane potential were detected exclusively during disruptions in the cell cycle, apoptosis, or necrosis. Typically, a depolarized mitochondrial membrane signifies that the cell is in the initial phases of apoptosis [53].

The percentages of mitochondrial membrane depolarization were found to be 27.1±3.2%, 54.1±6.5%, and 28.5±4.7%, respectively, after A549 cells were treated with carboplatin (100 µM), curcumin (60 µM), and a combined usage of curcumin+carboplatin (24 µM+40 µM) for 12 hours. Considering these results, a slight increase in depolarization was observed with the combination usage compared to carboplatin used alone, whereas there was a decrease in nearly half of the dose of carboplatin, which is toxic. When the duration was extended to 24 hours, no significant change was observed in the depolarization of curcumin (54.6±18.9%). However, carboplatin, which showed a decrease in depolarization, was determined as 16.8±0.0%. In the combination of Cur+Carbo (24 µM+40 µM), a slight increase was detected at 29.5±1.5%.

Özdemi *et al*. examined the changes in the mitochondrial membrane in order to examine the apoptotic effect mechanisms of cisplatin, resveratrol, and their combination in breast cancer cells. It was observed that the combined application of cisplatin and resveratrol resulted in higher membrane depolarization compared to the treatment alone. Simultaneously, the study found that the dosage of cisplatin-treated alone was dramatically decreased when resveratrol and cisplatin were used together [54].

When certain signals direct cells to go through apoptosis, the cell goes through a number of unique modifications. Early in apoptosis, a class of proteins called caspases is usually activated. Additionally, caspases can trigger cleavage enzymes like DNases [49].

In our study, the role of caspases 3 and 9, which play a crucial role in apoptosis, was investigated using flow cytometry. After treating A549 cells with carboplatin (100 µM), curcumin (60 µM), and combined use of curcumin+carboplatin (24 µM+40 µM) for 12 hours, the caspase 9 activity in each case was determined to be 10.6±2.0%, 8.31±0.8%, and 10.3±1.4%, respectively (Fig. **4** and Table **4**). No significant change was observed in caspase 9 activity between the carboplatin treatment and the combination. However, the combination treatment, by decreasing the toxic dose of carboplatin, demonstrated the same effect. This indicates that curcumin is effective in increasing caspase-9 activation. After treating A549 cells with an IC_50_ dose of carboplatin (100 µM) and IC_50_ dose of curcumin (60 µM) for 24 hours, the caspase 3 activity was determined to be 0.5±0.1% and 1.9±0.0%. When curcumin and carboplatin were administered in combination, caspase 3 activity significantly increased compared to the application of each drug alone, with a measured value of 7.3±0.8% (Table **5**). These results are consistent with the literature indicating that caspase 3 and 9 expression levels increase when carboplatin and curcumin are used in combination in A549 cells [48].

## CONCLUSION

In conclusion, the combination of curcumin and carboplatin demonstrated a synergistic effect in A549 cells. The combined treatment resulted in a significantly higher rate of early apoptotic cells compared to individual treatments, indicating an enhanced therapeutic effect. The success of this study lies in showing how a natural compound can potentiate the effects of a conventional chemotherapeutic agent. Additionally, when compared to carboplatin alone, the combined treatment exhibited increased mitochondrial membrane depolarization. The novelty of the study lies in the combined treatment of curcumin and carboplatin on the A549 cancer cell line, where key mechanisms, such as mitochondrial depolarization and caspase activation, serve as a foundation for future therapies. Although there was a slight increase in caspase 9 activity with the combined treatment, caspase 3 activity was notably elevated, suggesting enhanced apoptotic signaling pathways. These results suggest that the combined use of curcumin and carboplatin holds promise for reducing the toxic dose of carboplatin while enhancing its therapeutic efficacy.

## Figures and Tables

**Table 1:** The % inhibition and combination index of curcumin, carboplatin, and curcumin+carboplatin combination.

**IC_50_%**	**Concentration of Curcumin+Carboplatin**	**Combination Index**	**Inhibition%**
100%	60 µM Curcumin +100 µM Carboplatin	0.4663	84.9
75%	45 µM Curcumin +75 µM Carboplatin	0.4151	82.0
50%	30 µM Curcumin +50 µM Carboplatin	0.7209	58.6
40%	24 µM Curcumin+40 µM Carboplatin	0.7967	48.9
30%	18 µM Curcumin+30 µM Carboplatin	0.7897	30.9
20%	12 µM Curcumin +20 µM Carboplatin	1.1138	28.1
10%	6 µM Curcumin +10 µM Carboplatin	0.6537	18.3

**Table 2:** The percentage distribution of A549 cell apoptosis after 12 hours of incubation in different areas. The experiment was independently repeated three times. Data are presented as mean ± S.E.M.

**-**	**Viable**	**Early Apoptosis**	**Late Apoptosis**	**Necrosis**
Control	92.2±0.8	1.5±0.3	5.6±0.8	0.8±0.2
Curcumin (60 µM)	53.3±0.8	8.1±0.3	33.3±0.8	5.3±1.5
Carboplatin (100 µM)	46.2±4.1	9.5±1.3	38.5±3.3	5.9±2.3
Curcumin+Carboplatin (24 µM+40 µM)	38.4±7.2	22.2±2.9	36.6±6.2	2.8±1.1
Curcumin+Carboplatin (18 µM+30 µM)	45.0±1.5	9.0±6.9	41.7±5.7	4.3±2.2

**Table 3:** The percentage distribution of A549 cell apoptosis after 6 hours of incubation in different areas. The experiment was independently repeated three times. Data are presented as mean ± S.E.M.

**-**	**Viable**	**Early Apoptosis**	**Late Apoptosis**	**Necrosis**
Control	95.7±0.5	1.2±0.1	2.3±0.3	0.9±0.1
Curcumin (60 µM)	74.2±4.1	9.2±0.7	13.4±2.4	3.3±1.0
Carboplatin (100 µM)	95.5±0.1	1.3±0.1	2.6±0.1	0.6±0.1
Curcumin+Carboplatin (24 µM+40 µM)	95.0±0.2	2.0±0.1	2.6±0.0	0.6±0.0
Curcumin+Carboplatin (18 µM+30 µM)	93.6±0.6	2.5±0.4	3.6±0.2	0.5±0.0

**Table 4:** Effect of curcumin, carboplatin, and combined treatment on mitochondrial membrane potential in A549 cells for 12 and 24 hours.

**-**	**12 h**		**24 h**	
	**Intact ΔΨm**	**Fold ΔΨm**	**Intact ΔΨm**	**Fold ΔΨm**
Control	87.5±0.1	12.5±0.0	89.5±0.9	10.4±0.8
Curcumin (60 µM)	45.3±6.5	54.1±6.5	45.7±19.4	54.6±18.9
Carboplatin (100 µM)	72.7±3.2	27.1±3.2	83.5±0.1	16.8±0.0
Curcumin+Carboplatin (24 µM+40 µM)	71.1±4.7	28.5±4.7	70.6±1.4	29.5±1.5
Curcumin+Carboplatin (18 µM+30 µM)	71.9±1.9	27.3±2.3	78.0±2.1	22.0±2.1

**Note:** Data refer to the percentage of cells with intact ΔΨm (polarize) and fold ΔΨm (depolarize). The experiment was repeated 3 times independently. The data are presented as average ± S.E.M.

**Table 5:** The percentage of caspase-9 activity of A549 cells after curcumin, carboplatin, and curcumin+carboplatin treatment.

**-**	**12 h**		**24 h**	
	**Viable %**	**Caspase-9 Activity %**	**Viable %**	
Control	97.4±0.2	2.9±0.3	97.4±0.0	2.5±0.0
Curcumin (60 µM)	92.1±0.9	8.3±0.8	91.7±0.9	8.1±0.8
Carboplatin (100 µM)	89.7±1.9	10.6±2.0	94.4±0.1	5.6±0.0
Curcumin+Carboplatin (24 µM+40 µM)	90.1±1.4	**10.3±1.4**	96.4±0.1	3.6±0.2
Curcumin+Carboplatin (18 µM+30 µM)	90.9±0.4	9.2±0.4	96.6±0.3	3.4±0.3

**Note:** The experiment was repeated 3 times independently. The data are presented as average ± S.E.M and the value highlighted in bold indicates that the combination treatment achieves the same effect while reducing the toxic dose of carboplatin.

**Table 6:** Percentage of caspase-3 activity of A549 cells after curcumin, carboplatin, and curcumin+carboplatin treatment.

**-**	**12 h**		**24 h**	
	**Viable %**	**Caspase-3 Activity %**	**Viable %**	**Caspase-3 Activity %**
Control	99.4±0.1	0.5±0.1	98.6±0.6	1.2±0.6
Curcumin (60 µM)	98.2±0.5	1.6±0.4	97.8±0.0	1.9±0.0
Carboplatin (100 µM)	99.3±0.3	0.7±0.3	99.4±0.1	0.5±0.1
Curcumin+Carboplatin (24 µM+40 µM)	98.3±0.1	1.6±0.1	90.6±1.4	7.3±0.8
Curcumin+Carboplatin (18 µM+30 µM)	97.6±0.0	2.1±0.0	97.2±0.4	2.2±0.4

**Note:** The experiment was repeated 3 times independently. The data are presented as average ± S.E.M.

## Data Availability

All data generated or analyzed during this study are included in this published article.

## LIST OF ABBREVIATIONS

CI: Combination Index
NSCLC: Non-Small Cell Lung Cancer
PI: Propidium Iodide
